# Efficacy of Treatments in Reducing Facial Erythema in Rosacea: A Systematic Review

**DOI:** 10.1177/12034754241287546

**Published:** 2024-10-31

**Authors:** Nicholas J. Hua, Jennifer Chen, Ryan S. Q. Geng, Ronald Gary Sibbald, Cathryn Sibbald

**Affiliations:** 1 Temerty School of Medicine, University of Toronto, Toronto, ON, Canada; 2 Dalla Lana School of Public Health & Division of Dermatology, Department of Medicine, University of Toronto, Toronto, ON, Canada; 3 Division of Pediatric Dermatology, The Hospital for Sick Children, University of Toronto, Toronto, ON, Canada

**Keywords:** rosacea, erythema, treatment

## Abstract

Rosacea is a chronic inflammatory skin condition that affects over 5% of individuals worldwide. Its clinical presentation is characterized by an array of features, including erythema, papules and pustules, phymatous changes, telangiectasia, and ocular manifestations. Specifically, the multifaceted manifestation of erythema varies widely in intensity and distribution. Factors contributing to pathogenesis include neurovascular dysregulation, increased levels of pro-inflammatory mediators, and aberrant vasodilation. Erythema management plays an important role in reducing the psychosocial burden associated with rosacea and improving overall quality of life. Cochrane CENTRAL, Medline, and Embase databases were searched from inception to September 2023 and included 33 clinical trials reporting on a total of 7411 rosacea patients (74.1% female) and 21 different topical or systemic treatments. The mean age was 48.8 years (range, 18-83 years), and the mean time to outcome assessment was 8.1 weeks (standard deviation, 4.1 weeks). Treatment efficacy was assessed by outcome measures including percent improvement from baseline on 4- and 5-point scales, clinician erythema assessment (CEA) success (improvement ≥1 point), and CEA and patient self-assessment success (improvement ≥1 point). Pooled effect sizes for each treatment were calculated as a weighted average based on the number of patients in each study. The most effective topical treatments for reducing erythema include sodium sulphacetamide and sulphur, praziquantel, metronidazole, and B244 spray (*Nitrosomonas eutropha*). The most effective systemic treatment was paroxetine. Our findings highlight the varying efficacy of treatments in addressing the erythema in rosacea, recognizing the nuances of clinical presentations.

## Introduction

Rosacea is a common chronic inflammatory skin condition that affects more than 5% of the global population.^1,2^ It is characterized by major features including facial flushing, persistent erythema, papules, pustules, telangiectasia, and phymatous changes.^1,3^ In addition to the cutaneous features, ocular manifestations may also arise in up to 58% of patients.^1^ It is important to note that the clinical presentation of rosacea is heterogeneous, where patients may present with different combinations of clinical features.

The erythema associated with rosacea in particular represents a complex clinical challenge and likely has a multifactorial etiology. Contributing factors include neurovascular dysregulation, increased levels of pro-inflammatory mediators, and aberrant vasodilation.^1,4-6^ The presence of persistent erythema in the central facial region may have a marked impact on the patient’s body image, resulting in psychological distress and comorbidities.^7^ Many patients experience heightened self-consciousness and diminished self-esteem due to their visible facial symptoms, which can result in social anxiety, withdrawal from social interactions, and an overall decreased quality of life.^7,8^ The psychosocial impact of erythema underscores the importance of effective treatment and support for patients with rosacea.

A variety of therapies are currently available for treating rosacea, including topical, oral, and light-based modalities.^9^ The light-based therapies in rosacea are designed to target erythema (e.g., Pulsed dye laser, intense pulsed light), but these treatments are expensive, not covered by insurance, and yield temporary results. In addition, patients requiring access to clinics equipped with these treatment modalities typically need several sessions to achieve satisfactory clinical effect.^10^ Pharmaceutical treatment options are generally more accessible and have the additional benefit of being effective in reducing inflammatory lesions, a common feature observed in rosacea.^11^ With consideration of the psychosocial distress rosacea patients experience, the objective of this systematic review is to determine the efficacy of topical and systemic treatments for reducing erythema in rosacea.

## Methods

### Literature Search

The literature search for this systematic review (PROSPERO CRD42023463880) was conducted in accordance with the Preferred Reporting Items for Systematic Reviews and Meta-analyses (PRISMA) guidelines.^12^ Medline and Embase were searched using the following term combinations: rosacea and erythema, resulting in a total of 692 articles. The databases were searched from inception to September 2023, with no exclusions on language and patient demographics. The removal of 306 duplicates resulted in a total of 415 articles for screening. A PRISMA flow diagram is provided (Supplemental Figure 1). Full search strategies are provided in the supplemental material.

### Inclusion and Exclusion Criteria

All published articles reporting on the change in erythema in rosacea patients after treatment with any topical or systemic pharmaceutical treatment within a 4- to 16-week timepoint were included. Animal studies and articles that do not provide quantitative data on the change in erythema post-treatment were excluded.

### Data Extraction and Analysis

Articles were screened based on titles and abstract content, followed by full-text screening to determine final eligibility. Data extraction was performed by NJH and JC individually, with disagreements mediated by a third author. Treatment efficacy was assessed by outcome measures including percent improvement from baseline on 4- and 5-point scales, clinician erythema assessment (CEA) success (improvement ≥1 point), and CEA and patient self-assessment (PSA) success (improvement ≥1 point). Pooled effect sizes for each treatment were calculated as a weighted average based on the number of patients in each study. The National Institute of Health quality assessment tool was used to assess methodological quality, which provides quality ratings of “good,” “fair,” or “poor.” Only reports with a “good” rating were included.

## Results

**Figure 1.**
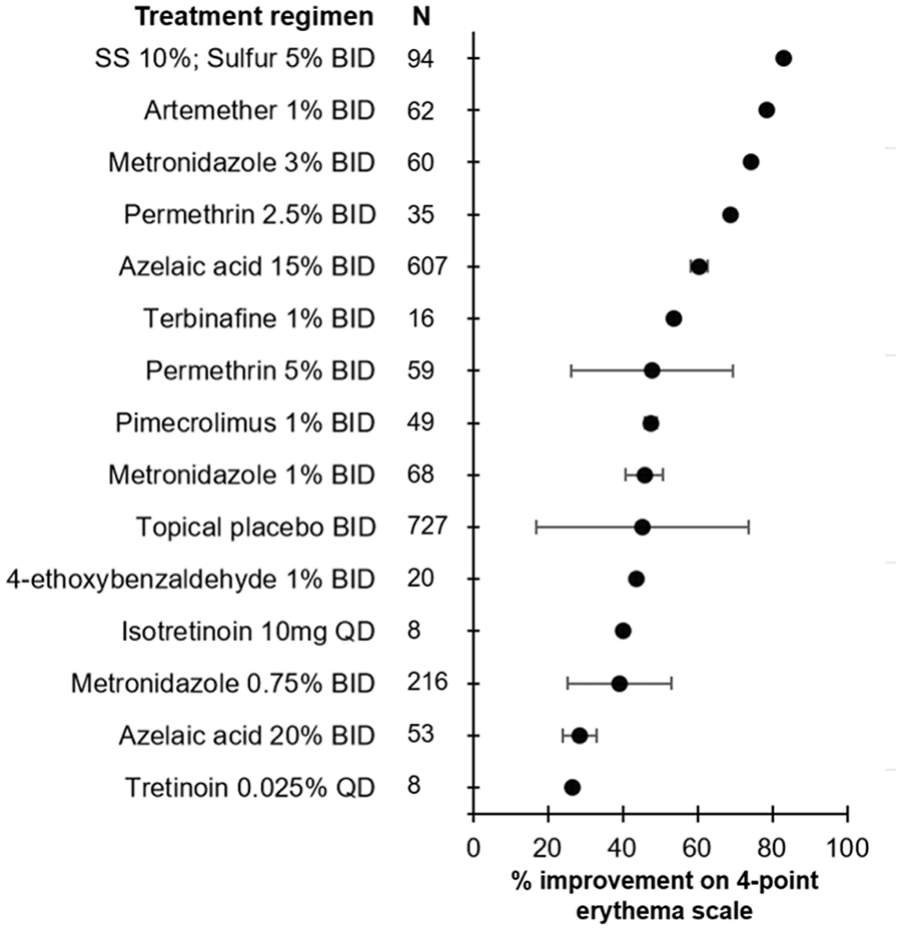
Efficacy of treatment regimens in improving erythema when measured on a 4-point scale in rosacea patients. The mean percent improvement in erythema score is represented by the bullet for each treatment. Bars represent standard deviations. SS, sodium sulphacetamide.

**Figure 2.**
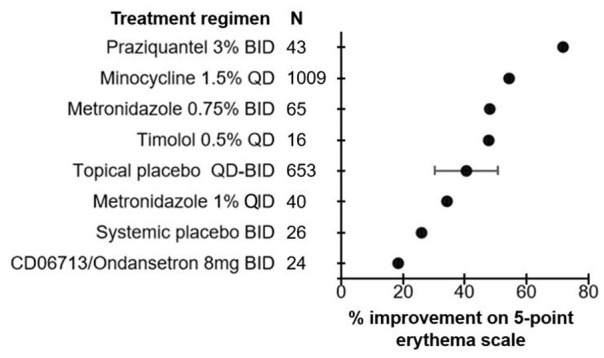
Efficacy of treatment regimens in improving erythema when measured on a 5-point scale in rosacea patients. The mean percent improvement in erythema score is represented by the bullet for each treatment. Bars represent standard deviations.

**Figure 3.**
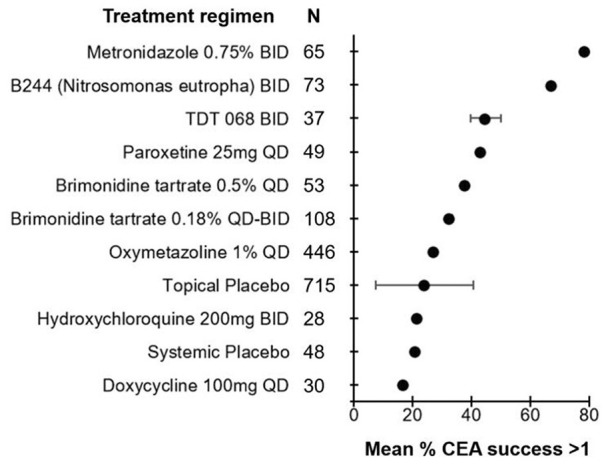
Efficacy of treatment regimens in improving erythema when measured in terms of CEA success (≥1 point improvement) in rosacea patients. The mean percent of patients achieving CEA success is represented by the bullet for each treatment. Bars represent standard deviations. CEA, clinician erythema assessment.

**Figure 4.**
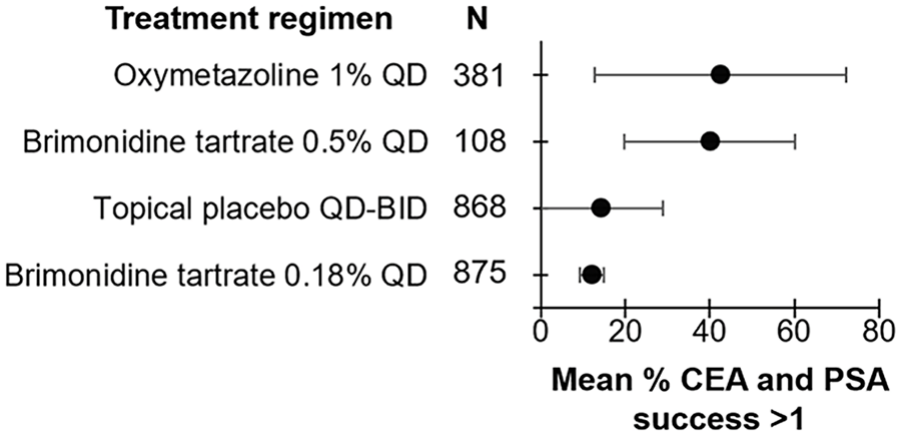
Efficacy of treatment regimens in improving erythema when measured in terms of CEA and PSA success (≥1 point improvement) in rosacea patients. The mean percent of patients achieving CEA and PSA success is represented by the bullet for each treatment. Bars represent standard deviations. CEA, clinician erythema assessment; PSA, patient self-assessment.

A total of 415 unique articles were identified with the outlined search strategy (Supplemental Figure 1). Of the 33 articles included, all were Randomized Control Trials (RCTs) with the exception of 1 uncontrolled trial, reporting on 7411 rosacea patients (74.1% female) and 21 different topical or systemic treatments (Supplementary Table 1). Supplemental tables summarizing the efficacy of treatments in reducing erythema are provided (Supplemental Tables 1–4). Diagrams ranking the treatments by percent post-treatment erythema improvement are also provided (Figures 1-4).

### Erythema Assessed by 4-Point Scale

#### 4-Ethoxybenzaldehyde [20 patients]

4-Ethoxybenzaldehyde was reported by 1 study in a 1% topical formulation applied twice daily.^13^ There was a mean 43.7% improvement in the 4-point scale score among the 20 patients, representing a 0.97-fold change compared to the topical placebo.

#### Artemether [62 patients]

Artemether was reported by 1 study in a 1% topical formulation applied twice daily.^14^ There was a mean 78.4% improvement in the 4-point scale score among the 62 patients, representing a 1.73-fold change compared to the topical placebo.

#### Azelaic acid [130 patients]

Azelaic acid was reported by 4 studies in either 15% or 20% topical formulations applied twice daily.^15-18^ For the 2 studies reporting on the 20% formulation, there was a mean 28.5% improvement in the 4-point scale score among the 53 patients, representing a 0.63-fold change compared to the topical placebo.^15,16^ For the remaining 2 studies reporting on the 15% formulation, there was a mean 60.4% improvement in the 4-point scale score among the 607 patients, representing a 1.34-fold change compared to the topical placebo.^17,18^

#### Isotretinoin [8 patients]

Isotretinoin was reported in 1 uncontrolled trial at a dose of 10 mg once daily. There was a mean 40.0% improvement in the 4-point scale score among the 8 patients.^19^

#### Metronidazole [344 patients]

Metronidazole was reported by 8 studies in 3%, 1%, or 0.75% formulations applied twice daily.^14,15,16,18,20-23^ For the 1 study reporting on the 3% formulation, there was a mean 74.4% improvement in erythema measured on a 4-point scale among 60 patients, representing a 1.64-fold change compared to topical placebo.^14^ For the 2 studies reporting on the 1% formulation, there was a mean 45.7% improvement in erythema measured on a 4-point scale among 68 patients, representing a 1.01-fold change compared to topical placebo.^15,16^ For the 5 studies reporting on the 0.75% formulation, there was a mean 39.2% improvement in erythema measured on a 4-point scale among the 216 patients, representing a 0.87-fold change compared to the topical placebo.^18,20-23^

#### Permethrin [94 patients]

Permethrin was reported by 4 studies in either 5% or 2.5% topical formulations applied twice daily.^16,22,24,25^ For the 3 studies reporting on the 5% formulation, there was a mean 47.8% improvement in the 4-point scale score among the 59 patients, representing a 1.06-fold change compared to the topical placebo.^16,22,24^ For the 1 study reporting on the 2.5% formulation, there was a mean 68.9% improvement in the 4-point scale score among the 35 patients, representing a 1.52-fold change compared to the topical placebo.^25^

#### Pimecrolimus [49 patients]

Pimecrolimus was reported by 2 studies in a 1% topical formulation applied twice daily.^21,26^ There was a mean 47.6% improvement in the 4-point scale score among the 49 patients, representing a 1.05-fold change compared to the topical placebo.

#### Sodium sulphacetamide/sulphur [94 patients]

Sodium sulphacetamide 10%/sulphur 5% topical formulation applied twice daily was reported by 1 study.^27^ There was a mean 83.0% improvement in the 4-point scale score among the 94 patients, representing a 1.84-fold change compared to the topical placebo.

#### Terbinafine [16 patients]

Terbinafine was reported by 1 study in a 1% topical formulation applied twice daily.^23^ There was a mean 53.7% improvement in the 4-point scale score among the 16 patients, representing a 1.19-fold change compared to the topical placebo.

#### Tretinoin [8 patients]

Tretinoin was reported by 1 study in a 0.025% topical formulation applied once daily.^19^ There was a mean 26.7% improvement in the 4-point scale score among the 8 patients, representing a 0.59-fold change compared to the topical placebo.

### Erythema Assessed by 5-Point Scale

#### CD06713 (Ondansetron) [24 patients]

CD06713 was reported by 1 study at a dose of 8 mg twice daily.^28^ There was a mean 18.4% improvement in the 5-point scale score among the 20 patients, representing a 0.70-fold change compared to the topical placebo.

#### Metronidazole [105 patients]

Metronidazole was reported by 2 studies in both 1% or 0.75% formulations applied once daily or twice daily.^29,30^ For the 1 study reporting on the 1% formulation, there was a mean 34.2% improvement in erythema measured on a 5-point scale among 65 patients, representing a 0.82-fold change compared to topical placebo.^29^ For the 1 study reporting on the 0.75% formulation, there was a mean 48.2% improvement in erythema measured on a 5-point scale among the 65 patients, representing a 1.15-fold change compared to the topical placebo.^30^

#### Minocycline [1009 patients]

Minocycline was reported by 1 study in a 1.5% topical formulation applied once daily.^31^ There was a mean 54.4% improvement in the 5-point scale score among the 1009 patients, representing a 1.30-fold change compared to the topical placebo.

#### Praziquantel [43 patients]

Praziquantel was reported by 1 study in a 3% topical formulation applied twice daily.^32^ There was a mean 71.9% improvement in the 5-point scale score among the 43 patients, representing a 1.72-fold change compared to the topical placebo.

#### Timolol [16 patients]

Timolol was reported by 1 study in a 0.5% topical formulation applied once daily.^33^ There was a mean 47.6% improvement in the 5-point scale score among the 16 patients, representing a 1.14-fold change compared to the topical placebo.

### Erythema Assessed by CEA Success (≥1 Point Improvement)

#### B244 (*Nitrosomonas eutropha*) [73 patients]

B244 was reported by 1 study in a 4 × 10E9 cells/ml topical formulation applied twice daily.^34^ Of the 73 patients, 67.1% achieved CEA success, representing a 2.79-fold change compared to the topical placebo.

#### Brimonidine tartrate [161 patients]

Brimonidine tartrate was reported by 1 study in both 0.5% and 0.18% topical formulations applied once daily or twice daily.^35^ There was a mean 37.7% rate of CEA success among 53 patients, representing a 1.57-fold improvement over topical placebo. The study also reported on the 0.18% formulation, noting a mean 32.4% rate of CEA success among 108 patients, representing a 1.35-fold improvement compared to the topical placebo.

#### Doxycycline [30 patients]

Doxycycline was reported in 1 study at a dose of 100 mg once daily.^36^ There was a mean 16.7% rate of CEA success among 108 patients, representing a 0.80-fold change compared to systemic placebo.

#### Hydroxychloroquine [28 patients]

Hydroxychloroquine was reported in 1 study at a dose of 200 mg twice daily.^36^ There was a mean 21.4% rate of CEA success among 28 patients, representing a 1.03-fold change compared to the systemic placebo.

#### Metronidazole [65 patients]

Metronidazole was reported in 1 study in 0.75% of formulations applied once daily or twice daily.^30^ There was a mean 78.5% rate of CEA success among the 65 patients, representing a 3.26-fold change compared to the topical placebo.

#### Oxymetazoline [446 patients]

Oxymetazoline was reported in 1 study in a 1% topical formulation applied once daily.^37^ There was a mean 27.1% rate of CEA success among 446 patients, representing a 1.13-fold change compared to the topical placebo.

#### Paroxetine [49 patients]

Paroxetine was reported in 1 study at a dose of 25 mg once daily.^36^ There was a mean 42.9% rate of CEA success among 446 patients, representing a 2.06-fold change compared to the systemic placebo.

#### TDT 068 [37 patients]

TDT 068 was reported in 1 study in a topical formulation applied twice daily.^38^ There was a mean 44.8% rate of CEA success among the 37 patients, representing a 1.86-fold change compared to the topical placebo.

### Erythema Assessed by CEA and PSA Success (≥1 Point Improvement)

#### Brimonidine tartrate [489 patients]

Brimonidine tartrate was reported by 4 studies in either 0.5% or 0.18% topical formulations applied once daily or twice daily.^35,39-41^ For the 4 studies reporting on the 0.5% formulation, there was a mean 39.9% rate of CEA and PSA success among the 381 patients, representing a 2.81-fold change compared to the topical placebo.^35,39-41^ For the study additionally reporting on the 0.18% formulation, there was a mean 12.1% rate of CEA and PSA success among the 108 patients, representing a 0.85-fold change compared to the topical placebo.^35^

#### Oxymetazoline [868 patients]

Oxymetazoline was reported in 3 studies in 1% topical formulations applied once daily.^42-44^ For the 3 studies reporting on the 1% formulation, there was a mean 42.5% rate of CEA and PSA success among the 868 patients, representing a 2.98-fold change compared to the topical placebo.

## Discussion

The therapeutic efficacy of 21 different topical or systemic therapies for improving erythema was assessed in 33 articles, covering a total of 7415 rosacea patients. All articles included were RCTs, with the exception of 1 uncontrolled trial.

Topical treatments generally achieved greater improvements in erythema reduction measured in both 4- and 5-point scales and CEA and CEA + PSA success rates over placebo compared to systemic treatments. The only exception was paroxetine, with a 2.06-fold improvement over the systemic placebo when assessed by CEA success. It should be noted that systemic isotretinoin was only given as a low-dose regimen of 10 mg once daily.

Paroxetine achieved the highest rate of CEA success among systemic treatments. Paroxetine may reduce flushing by decreasing blood flow to the skin, thus demonstrating efficacy against the vasodilatory aspect of erythema in rosacea.^45,46^ Moreover, paroxetine has been shown to inhibit the production of pro-inflammatory cytokines such as interleukin 6 (IL-6) and interleukin 1β (IL-1β), which is elevated in rosacea patients.^47,48^ In addition, paroxetine may promote the production of inducible nitric oxide synthase and nitric oxide which have anti-inflammatory effects.^48^ Paroxetine also regulates complex inflammatory pathways including nuclear factor kappa-light-chain-enhancer of activated B cells, inflammasomes, toll-like receptor 4, and peroxisome proliferator-activated receptor gamma.^49^ These pathways are critical components of the immunological response, and their modulation can help reduce inflammation and subsequent erythema in rosacea patients.^49^ Paroxetine’s more common side effects include disturbances of sleep, drowsiness, loss of appetite, sweating, and sexual side effects.^50^ It also functions as an anti-depressant and may potentially interact with other medications.^50^

Among the topical treatments, sodium sulphacetamide 10%/sulphur 5% and praziquantel at a 3% dosage were the most effective at improving erythema when measured on a 4-point and 5-point scale, respectively. Sodium sulphacetamide works by blocking bacterial dihydropteroate synthetase, preventing p-aminobenzoic acid from being converted to folic acid.^51,52^ This mechanism inhibits the growth of various gram-negative and gram-positive organisms, including *Propionibacterium acnes.*^52^ Sulphur can also help reduce the production of sebum which may have pro-inflammatory effects and contribute to the development of erythema.^53^ In addition, it demonstrates beneficial keratolytic effects by promoting dead skin cell exfoliation, assisting in the removal of surface contaminants, and leading to a smoother and more refined skin texture.^52^ Sulphacetamide-sulphur side effects typically present as dryness, erythema, itchiness, and general discomfort.^54^

Praziquantel has been suggested to have immunomodulatory effects, possibly by influencing cytokine production and immune cell activity, specifically macrophages, causing an anti-inflammatory effect.^55-57^

In terms of CEA success, metronidazole 0.75% and B244 (*Nitrosomonas eutropha*) applied as a 4 × 10E9 cells/ml topical formulation were the most effective in improving erythema. Metronidazole, also the most commonly studied topical treatment, acts by scavenging reactive oxygen species, inhibiting pro-inflammatory cytokine production, and displaying bactericidal activity against *Bacillus oleronius.*^58,59^ This organism has been implicated in eliciting an inflammatory response in 73% of individuals with rosacea.^60,61^ Ammonia and urea from sweat are converted by *Nitrosomonas eutropha* into nitrite, which has antibacterial qualities, and nitric oxide, which modulates inflammation and vasodilation.^62^ Topical metronidazole has common side effects such as burning, dryness, itching, and stinging.^63^ *Nitrosomonas eutropha*’*s* most common side effects include gastrointestinal disorders, headaches, infections/infestations, and musculoskeletal and connective tissue disorders.^64^

The study is subject to certain limitations, such as the heterogeneous patient population and sample sizes. It is important to note that the specific skin colour of patients was not consistently specified across included studies, and variations in baseline pigmentation may introduce a confounding factor in the accurate assessment of erythema improvement following treatment interventions. Moreover, the breadth of understanding may have been limited by limiting the scope to quantitative data on the change in erythema, which may have missed important qualitative insights or anecdotal material regarding treatment experiences. The use of different scales in assessing erythema and timing endpoints may limit the ability to compare all treatments included in this study. A consensus on the method of assessing erythema in clinical trials is required.

## Conclusion

This systematic review comprehensively assessed the efficacy of various treatments in reducing erythema associated with rosacea, recognizing the imperative need for effective interventions to alleviate both the physical and psychosocial burden affecting over 5% of individuals worldwide. The findings underscore the multifaceted nature of rosacea, wherein the intricate interplay of vascular, inflammatory, and neurovascular mechanisms contributes to the development of erythema. The diverse array of treatments examined in this review, ranging from topical agents to laser therapies, has demonstrated varying degrees of success in addressing this challenging symptom. The most well-studied treatments addressing erythema in rosacea include metronidazole, azelaic acid, permethrin, oxymetazoline, and brimonidine tartrate. Sodium sulphacetamide and sulphur, praziquantel, metronidazole, and B244 (*Nitrosomonas eutropha*) emerge as the most effective treatments in reducing erythema associated with rosacea, showcasing their notable efficacy in targeting the underlying inflammatory and microbial factors that contribute to the condition.

## Supplemental Material

sj-docx-1-cms-10.1177_12034754241287546 – Supplemental material for Efficacy of Treatments in Reducing Facial Erythema in Rosacea: A Systematic Review Supplemental material, sj-docx-1-cms-10.1177_12034754241287546 for Efficacy of Treatments in Reducing Facial Erythema in Rosacea: A Systematic Review by Nicholas J. Hua, Jennifer Chen, Ryan S. Q. Geng, Ronald Gary Sibbald and Cathryn Sibbald in Journal of Cutaneous Medicine and Surgery
