# Supplementary material for: Efficacy of Treatments in Reducing Facial Erythema in Rosacea: A Systematic Review
Source: J Cutan Med Surg. 2024 Oct 31;29(1):43–50. doi: 10.1177/12034754241287546 (PMC11829502; doi:10.1177/12034754241287546)
Supplement: sj-docx-1-cms-10.1177_12034754241287546 – Supplemental material for Efficacy of Treatments in Reducing Facial Erythema in Rosacea: A Systematic Review [file sj-docx-1-cms-10.1177_12034754241287546.docx]

**Supplemental Material**

**Search strategy**

Medline and Embase

1 rosacea.ab,ti.

2 erythema.ab,ti.

3 redness.ab,ti.

4 flush.ab,ti.

5 blush.ab,ti.

6 exp rosacea/

7 2 or 3 or 4 or 5

8 1 or 6

9 7 and 8

10 limit 9 to (clinical trial or randomized controlled trial or controlled clinical trial or multicenter study or phase 1 clinical trial or phase 2 clinical trial or phase 3 clinical trial or phase 4 clinical trial)

11 limit 10 to humans

**Cochrane central**

Rosacea and erythema

**Supplementary Table 1.** Studies included in this review showing treatment, sample size, mean age including standard deviation or range, male population percentage, erythema assessment and time-point data.

| **Article** | **Treatment regimen** | **N patients** | **Mean age (SD/range)** | | **% Male** | | **Erythema assessment method** | **%improvement or %patient achieving success** | **Time point (weeks)** |
| --- | --- | --- | --- | --- | --- | --- | --- | --- | --- |
| Bribeche 2015[^1^](https://paperpile.com/c/PjMad2/M1C5) | Praziquantel 3% BID  Placebo | 43  22 | | 44.8 (11.2)  39.6 (10.6) | | 48.8  50.0 | 5-point scale  5-point scale | 71.9  28.8 | 16 weeks  16 weeks |
| ChiCTR-IPR-17012224 2017[^2^](https://paperpile.com/c/PjMad2/mtjF) | Doxycycline 100mg QD  Hydroxychloroquine 200 mg BID | 30  28 | | 34.8 (11.2)  32.7 (10.0) | | 10.0  90.0 | CEA success  CEA success | 16.7  21.4 | 4 weeks  4 weeks |
| Draelos 2005[^3^](https://paperpile.com/c/PjMad2/dnC0) | 4-ethoxybenzaldehyde 1% BID  Placebo | 20  10 | | NR | | NR | 4-point scale  4-point scale | 43.7  16.7 | 4 weeks  4 weeks |
| Draelos 2015[^4^](https://paperpile.com/c/PjMad2/cXcE) | Azelaic Acid 15% BID  Placebo | 483  478 | | 51.2 (12.3)  51.9 (13.2) | | 26.7  27.2 | 4-point scale  4-point scale | 61.5  51.3 | 12 weeks  12 weeks |
| Ebneyamin 2020[^5^](https://paperpile.com/c/PjMad2/NpwJ) | Permethrin 2.5% BID  Placebo | 35  35 | | NR | | NR | 4-point scale  4-point scale | 68.9  12.9 | 12 weeks  12 weeks |
| Elewski 2003[^6^](https://paperpile.com/c/PjMad2/eNPG) | Azelaic Acid 15% BID  Metronidazole 0.75% BID | 124  127 | | 49 (23-83)  46 (18-82) | | 32.3  33.9 | 4-point scale  4-point scale | 56.0  42.0 | 15 weeks  15 weeks |
| Ertl 1994[^7^](https://paperpile.com/c/PjMad2/Ciaz) | Isotretinoin 10 mg QD  Tretinoin 0.025% QD | 8  8 | | 59 (34-77)  59 (34-77) | | N/A  N/A | 4-point scale  4-point scale | 40.0  26.7 | 16 weeks  16 weeks |
| EUCTR2006-003707-40-DE 2006[^8^](https://paperpile.com/c/PjMad2/abni) | CD06713/Ondansetron  Placebo | 24  26 | | 47.2 (10.1)  46.8 (11.7) | | 16.7  15.4 | 5-point scale  5-point scale | 18.4  26.2 | 4 weeks  4 weeks |
| EUCTR2012-001044-22-SE 2012[^9^](https://paperpile.com/c/PjMad2/1HBf) | Brimonidine tartrate 0.5% QD  Placebo | 57  55 | | 43.2 (10.8)  44.9 (12.1) | | 28.1  27.3 | CEA and PSA success  CEA and PSA success | 68.4  60.0 | 4 weeks  4 weeks |
| Fowler 2013[^10^](https://paperpile.com/c/PjMad2/6db5) | Brimonidine tartrate 0.5% QD  Placebo | 129  131 | | 49.5 (11.8)  48.1 (12.8) | | 19.4  22.1 | CEA and PSA success  CEA and PSA success | 56.7  30.5 | 4 weeks  4 weeks |
| GamborgNielsen 1983[^11^](https://paperpile.com/c/PjMad2/DUId) | Metronidazole 1% QD  Placebo | 40  37 | | 47 (26-87)  47 (26-87) | | 39.5  39.5 | 5-point scale  5-point scale | 34.2  16.2 | 8 weeks  8 weeks |
| *Gold 2018[^12^](https://paperpile.com/c/PjMad2/cUCi) | Oxymetazoline 1% QD | 422 | | NR | | 23.9 | CEA and PSA success | 73.0 | 4 weeks |
| Gold 2020[^13^](https://paperpile.com/c/PjMad2/GI84) | Minocycline 1.5% QD  Minocycline 1.5% QD  Placebo  Placebo | 495  514  256  257 | | 48.9 (13.7)  50.9 (13.9)  49.7 (12.9)  50.9 (13.5) | | 28.3  29.0  27.3  34.6 | 5-point scale  5-point scale  5-point scale  5-point scale | 54.1  54.8  48.9  45.3 | 12 weeks  12 weeks  12 weeks  12 weeks |
| IRCT2014030416837N1 2014[^13,14^](https://paperpile.com/c/PjMad2/GI84+Wv44) | Permethrin 5% BID  Placebo | 20  20 | | NR | | NR | 4-point scale  4-point scale | 72.0  40.7 | 12 weeks  12 weeks |
| Karabulut 2008[^15^](https://paperpile.com/c/PjMad2/B515) | Pimecrolimus 1% BID  Placebo | 25  25 | | 46.8 (9.2)  46.8 (9.2) | | 12.0  12.0 | 4-point scale  4-point scale | 49.1  39.9 | 4 weeks  4 weeks |
| Koca 2010[^16^](https://paperpile.com/c/PjMad2/9UN2) | Metronidazole 1% BID  Pimecrolimus 1% BID | 24  24 | | 50.7 (9.1)  48.4 (9.4) | | 66.7  52.0 | 4-point scale  4-point scale | 52.6  46.0 | 12 weeks  12 weeks |
| Kocak 2002[^17^](https://paperpile.com/c/PjMad2/VoI2) | Metronidazole 0.75% BID  Permethrin 5% BID  Placebo | 20  23  20 | | 51 (20-80)  51 (20-80)  51 (20-80) | | 23.8  23.8  23.8 | 4-point scale  4-point scale  4-point scale | 50.9  48.5  1.9 | 8 weeks  8 weeks  8 weeks |
| Luger 2015[^18^](https://paperpile.com/c/PjMad2/EoLz) | TDT 068  TDT 068  Placebo  Placebo | 37  37  18  18 | | 52.0 (10.8)  52.0 (10.8)  51.3 (10.6)  51.3 (10.6) | | 20.0  20.0  23.8  23.8 | CEA success  CEA success  CEA success  CEA success | 50.0  39.5  35.0  14.0 | 4 weeks  4 weeks |
| Maddin 1999[^19^](https://paperpile.com/c/PjMad2/YHEV) | Azelaic Acid 20% BID  Metronidazole 0.75% BID | 37  37 | | 50.3 (26-75)  50.3 (26-75) | | 27.5  27.5 | 4-point scale  4-point scale | 25.5  18.7 | 15 weeks  15 weeks |
| Miyachi 2022[^20^](https://paperpile.com/c/PjMad2/UtJq) | Metronidazole 0.75% BID  Placebo | 65  65 | | 45.9 (10.6)  49.8 (14.9) | | 16.9  18.5 | 5-point scale  CEA success  5-point scale  CEA success | 48.2  78.5  25.3  56.9 | 12 weeks  12 weeks |
| Mostafa 2009[^21^](https://paperpile.com/c/PjMad2/dh37) | Azelaic Acid 20% BID  Metronidazole 0.75% BID  Permethrin 5% BID | 16  16  16 | | NR | | 4.2 | 4-point scale  4-point scale  4-point scale | 35.3  18.8  16.7 | 15 weeks  15 weeks  15 weeks |
| NCT01174030 2010^22^ | Brimonidine tartrate 0.5% QD  Brimonidine tartrate 0.18% QD  Brimonidine tartrate 0.18% BID  Placebo  Placebo | 53  54  54  55  53 | | 44.9 (11.5)  46.9 (12.7)  43.2 (12.3)  43.4 (12.7)  43.0 (12.3) | | 20.8  18.5  22.2  18.2  17.0 | CEA and PSA success  CEA success  CEA and PSA success  CEA success  CEA and PSA success  CEA success  CEA and PSA success  CEA success  CEA and PSA success  CEA success | 18.9  37.7  9.3  31.5  14.8  33.3  3.6  21.8  17.0  30.2 | 4 weeks  4 weeks  4 weeks  4 weeks  4 weeks  4 weeks  4 weeks  4 weeks  4 weeks  4 weeks |
| NCT01355471 2011[^2^](https://paperpile.com/c/PjMad2/zo8G)^3^ | Brimonidine tartrate 0.5% QD  Placebo | 142  142 | | 48.5 (11.9)  46.5 (12.1) | | 29.1  25.5 | CEA and PSA success  CEA and PSA success | 21.1  9.9 | 4 weeks  4 weeks |
| NCT02131636 2014[^2^](https://paperpile.com/c/PjMad2/nhRu)^4^ | Oxymetazoline 1% QD  Placebo | 222  218 | | NR  NR | | 22.5  19.7 | CEA and PSA success  CEA and PSA success | 14.9  6.0 | 4 weeks  4 weeks |
| NCT02132117 2014[^2^](https://paperpile.com/c/PjMad2/GqlU)^5^ | Oxymetazoline 1% QD  Placebo | 224  221 | | NR  NR | | 21.0  21.7 | CEA and PSA success  CEA and PSA success | 12.3  6.1 | 4 weeks  4 weeks |
| NCT03590366 2018[^26^](https://paperpile.com/c/PjMad2/Xn4o) | B244 (Nitrosomonas eutropha) 4x10E9 cells/ml BID  Placebo | 73  67 | | 50.6 (15.1)  52.4 (13.0) | | 24.7  28.4 | CEA success  CEA success | 67.1  56.7 | 8 weeks |
| Sauder 1997[^27^](https://paperpile.com/c/PjMad2/6pH7) | Sodium sulfacetamide 10%/Sulfur 5%  Placebo | 94  94 | | 50.0 (22-78)  50.0 (22-78) | | 38.8  38.8 | 4-point scale  4-point scale | 83.0  31.0 | 8 weeks  8 weeks |
| Serdar 2011[^28^](https://paperpile.com/c/PjMad2/B7iZ) | Metronidazole 0.75% BID  Terbinafine 1% BID | 16  16 | | 50.5 (11.6)  50.1 (15.0) | | 25.0  18.2 | 4-point scale  4-point scale | 70.4  53.7 | 8 weeks  8 weeks |
| Stein-Gold 2018[^29^](https://paperpile.com/c/PjMad2/D6Mz) | Oxymetazoline 1% QD  Placebo | 446  439 | | 49.9 (NR)  49.9 (NR) | | 21.3  21.3 | CEA success  CEA success | 27.1  13.7 | 4 weeks  4 weeks |
| Tan 2002[^30^](https://paperpile.com/c/PjMad2/rr4y) | Metronidazole 1% BID  Placebo | 44  45 | | 51.0 (1.7)  47.7 (1.5) | | 21.3  30.5 | 4-point scale  4-point scale | 42.0  27.0 | 12 weeks  12 weeks |
| Wang 2019[^31^](https://paperpile.com/c/PjMad2/w1B1) | Artemether 1%, BID  Metronidazole 3% BID | 62  60 | | 41.7 (21-60)  44.6 (23-61) | | 40.0  40.0 | 4-point scale  4-point scale | 78.4  74.4 | 4 weeks  4 weeks |
| Wang 2023[^32^](https://paperpile.com/c/PjMad2/JTVA) | Paroxetine 25 mg QD  Placebo | 49  48 | | 31.7 (10.9)  31.1 (10.1) | | 10.3  7.4 | CEA success  CEA success | 42.9  20.8 | 12 weeks  12 weeks |
| Wei 2021[^33^](https://paperpile.com/c/PjMad2/x8Qy) | Timolol 0.5% QD  Placebo | 16  16 | | 37.5 (22-47)  37.5 (22-47) | | 12.5  12.5 | 5-point scale  5-point scale | 47.6  16.0 | 4 weeks  4 weeks |

All studies included in this review were RCTs, except for 1 uncontrolled trial denoted by an asterisk. Abbreviations: CEA = clinician erythema assessment, PSA = patient self-assessment, NR = not reported.

**Supplemental Table 2.** Efficacy of treatment regimens in improving erythema when measured by improvement on a 4-point scale score in rosacea patients.

| **Treatment regimen** | **N patients** | **Mean Age (SD/range)** | **% Male** | **Mean % erythema improvement** | **Fold-improvement over placebo** |
| --- | --- | --- | --- | --- | --- |
| **Topical therapies** |  |  |  |  |  |
| Sodium sulfacetamide 10%; Sulfur 5% BID | 94 | 50.0 (14.0) | 38.8 | 83.0 | 1.84 |
| Artemether 1% BID | 62 | 41.7 (9.8) | 40.0 | 78.4 | 1.73 |
| Metronidazole 3% BID | 60 | 44.6 (9.5) | 40.0 | 74.4 | 1.64 |
| Permethrin 2.5% BID | 35 | NR | NR | 68.9 | 1.52 |
| Azelaic acid 15% BID | 607 | 49 (23-83) | 32.3 | 60.4 | 1.34 |
| Terbinafine 1% BID | 16 | 50.1 (15.0) | 18.2 | 53.7 | 1.19 |
| Permethrin 5% BID | 59 | 51 (20-80) | 13.1 | 47.8 | 1.06 |
| Pimecrolimus 1% BID | 49 | 47.6 (9.3) | 31.6 | 47.6 | 1.05 |
| Metronidazole 1% BID | 68 | 50.89 (5.6) | 37.3 | 45.7 | 1.01 |
| Topical Placebo BID | 727 | 51.1 (12.5) | 27.8 | 45.2 | - |
| 4-ethoxybenzaldehyde 1% BID | 20 | NR | NR | 43.7 | 0.97 |
| Metronidazole 0.75% BID | 216 | 47.7 (18-82) | 29.0 | 39.2 | 0.87 |
| Azelaic acid 20% BID | 53 | 50.3 (26-75) | 18.8 | 28.5 | 0.63 |
| Tretinoin 0.025% QD | 8 | 59 (34-77) | NR | 26.7 | 0.59 |
| **Systemic therapies** |  |  |  |  |  |
| Isotretinoin 10mg QD | 8 | 59 (34-77) | NR | 40.0 | - |

Abbreviations: NR = not reported

**Supplemental Table 3.** Efficacy of treatment regimens in improving erythema when measured by improvement on a 5-point scale score in rosacea patients.

| **Treatment regimen** | **N patients** | **Mean Age (SD/range)** | **% Male** | **Mean % erythema improvement** | **Fold-improvement over placebo** |
| --- | --- | --- | --- | --- | --- |
| **Topical therapies** |  |  | **Ta** |  |  |
| Praziquantel 3% BID | 43 | 44.8 (11.2) | 48.8 | 71.9 | 1.72 |
| Minocycline 1.5% QD | 1009 | 49.9 (13.8) | 28.7 | 54.4 | 1.30 |
| Metronidazole 0.75% BID | 65 | 45.9 (10.6) | 16.9 | 48.2 | 1.15 |
| Timolol 0.5% QD | 16 | 37.5 (22-47) | 12.5 | 47.6 | 1.14 |
| Topical Placebo QD-BID | 653 | 49.4 (13.3) | 30.4 | 41.8 | - |
| Metronidazole 1% QD | 40 | 47 (26-87) | 39.5 | 34.2 | 0.82 |
| **Systemic therapies** |  |  |  |  |  |
| Systemic Placebo BID | 26 | 46.8 (11.7) | 15.4 | 26.2 | - |
| CD06713/Ondansetron 8mg BID | 24 | 47.2 (10.1) | 16.7 | 18.4 | 0.70 |

**Supplemental Table 4.** Efficacy of treatment regimens in improving erythema when measured in terms of rosacea patients achieving CEA success (≥ 1 point improvement on CEA scales).

| **Treatment regimen** | | | **N patients** | **Mean Age (SD)** | **% Male** | **Mean % patients achieving CEA+PSA success** | **Fold-change over placebo** |
| --- | --- | --- | --- | --- | --- | --- | --- |
| **Topical therapies** |  | | |  | | | |
| Metronidazole 0.75% BID | | | 65 | 45.9 (10.6) | 16.9 | 78.5 | 3.26 |
| B244 (*Nitrosomonas eutropha*) 4x10E9 cells/ml BID | | | 73 | 48.5 (11.9) | 24.7 | 67.1 | 2.79 |
| TDT 068 BID | | | 37 | 52.0 (10.8) | 20.0 | 44.8 | 1.86 |
| Brimonidine tartrate 0.5% QD | | | 53 | 44.9 (11.5) | 20.8 | 37.7 | 1.57 |
| Brimonidine tartrate 0.18% QD-BID | | | 108 | 45.1 (12.5) | 20.4 | 32.4 | 1.35 |
| Oxymetazoline 1% QD | | | 446 | 49.9 (NR) | 21.3 | 27.1 | 1.13 |
| Topical Placebo QD-BID | | | 715 | 49.2 (13.0) | 21.3 | 24.1 | - |
| **Systemic therapies** | |  | |  | | | |
| Paroxetine 25mg QD | | | 49 | 31.7 (10.9) | 10.3 | 42.9 | 2.06 |
| Hydroxychloroquine 200mg BID | | | 28 | 32.7 (10.0) | 90.0 | 21.4 | 1.03 |
| Systemic Placebo | | | 48 | 37.5 (22-47) | 12.5 | 20.8 | - |
| Doxycycline 100mg QD | | | 30 | 34.8 (11.2) | 10.0 | 16.7 | 0.80 |

Abbreviations: NR = not reported

**Supplemental Table 5.** Efficacy of treatment regimens in improving erythema when measured in terms of rosacea patients achieving CEA and PSA success (≥ 1 point improvement on CEA and PSA scale).

| **Treatment regimen** | **N patients** | **Mean Age (SD)** | **% Male** | **Mean % patients achieving CEA success** | **Fold-change over placebo** |
| --- | --- | --- | --- | --- | --- |
| Oxymetazoline 1% QD | 868 | NR | 22.8 | 42.5 | 2.98 |
| Brimonidine tartrate 0.5% QD | 381 | 47.5 (11.7) | 24.5 | 39.9 | 2.81 |
| Topical Placebo QD-BID | 875 | 46.0 (12.4) | 21.7 | 14.2 | - |
| Brimonidine tartrate 0.18% QD-BID | 108 | 45.1 (12.5) | 20.4 | 12.1 | 0.85 |

Abbreviations: NR = not reported

**References**

1. [Bribeche MR, Fedotov VP, Gladichev VV, Pukhalskaya DM, Kolitcheva NL. Clinical and experimental assessment of the effects of a new topical treatment with praziquantel in the management of rosacea. Int J Dermatol. 2015 Apr;54(4):481–7.](http://paperpile.com/b/PjMad2/M1C5)

2. [Wang B, Yuan X, Huang X, Tang Y, Zhao Z, Yang B, et al. Efficacy and safety of hydroxychloroquine for treatment of patients with rosacea: A multicenter, randomized, double-blind, double-dummy, pilot study. J Am Acad Dermatol. 2021 Feb;84(2):543–5.](http://paperpile.com/b/PjMad2/mtjF)

3. [Draelos ZD, Fuller BB. Efficacy of 1% 4-ethoxybenzaldehyde in reducing facial erythema. Dermatol Surg. 2005 Jul;31(7 Pt 2):881–5; discussion 885.](http://paperpile.com/b/PjMad2/dnC0)

4. Draelos ZD, Elewski BE, Harper JC, Sand M, Staedtler G, Nkulikiyinka R, et al. [Randomized, phase III, double-blind, vehicle-controlled clinical trial to evaluate the safety and efficacy of 12 weeks of twice-daily azelaic acid foam, 15% in papulopustular rosacea. J Am Acad Dermatol. 2015 May;72(5):AB59.](http://paperpile.com/b/PjMad2/cXcE)

5. [Ebneyamin E, Mansouri P, Rajabi M, Qomi M, Asgharian R, Azizian Z. The efficacy and safety of permethrin 2.5% with tea tree oil gel on rosacea treatment: A double-blind, controlled clinical trial. J Cosmet Dermatol. 2020 Jun;19(6):1426–31.](http://paperpile.com/b/PjMad2/NpwJ)

6. [Elewski BE, Fleischer AB Jr, Pariser DM. A comparison of 15% azelaic acid gel and 0.75% metronidazole gel in the topical treatment of papulopustular rosacea: results of a randomized trial. Arch Dermatol. 2003 Nov;139(11):1444–50.](http://paperpile.com/b/PjMad2/eNPG)

7. [Ertl GA. A comparison of the efficacy of topical tretinoin and low-dose oral isotretinoin in Rosacea. Arch Dermatol. 1994 Mar 1;130(3):319.](http://paperpile.com/b/PjMad2/Ciaz)

8. [EudraCT Number 2006-003707-40 - Clinical trial results - EU Clinical Trials Register [Internet]. [cited 2023 Dec 14]. Available from:](http://paperpile.com/b/PjMad2/abni) <https://www.clinicaltrialsregister.eu/ctr-search/trial/2006-003707-40/results>

9. [EudraCT Number 2012-001044-22 - Clinical trial results - EU Clinical Trials Register [Internet]. [cited 2023 Dec 14]. Available from:](http://paperpile.com/b/PjMad2/1HBf) <https://www.clinicaltrialsregister.eu/ctr-search/trial/2012-001044-22/results>

10. [Fowler J, Jackson M, Moore A, Jarratt M, Jones T, Meadows K, et al. Efficacy and safety of once-daily topical brimonidine tartrate gel 0.5% for the treatment of moderate to severe facial erythema of rosacea: results of two randomized, double-blind, and vehicle-controlled pivotal studies. J Drugs Dermatol [Internet]. 2013 Jun 1 [cited 2023 Dec 14];12(6). Available from:](http://paperpile.com/b/PjMad2/6db5) <https://pubmed.ncbi.nlm.nih.gov/23839181/>

11. [Nielsen PG. Treatment of rosacea with i% metronidazole cream. A double-blind study. Br J Dermatol [Internet]. 1983 Mar [cited 2023 Dec 14];108(3). Available from:](http://paperpile.com/b/PjMad2/DUId) <https://pubmed.ncbi.nlm.nih.gov/6219689/>

12. [Gold MH, Lebwohl M, Biesman BS, Robinson DM, Luo L, Berk DR, et al. Daily oxymetazoline cream demonstrates high and sustained efficacy in patients with persistent erythema of rosacea through 52 weeks of treatment. J Am Acad Dermatol. 2018 Sep;79(3):e57–9.](http://paperpile.com/b/PjMad2/cUCi)

13. [Gold LS, Del Rosso JQ, Kircik L, Bhatia ND, Hooper D, Nahm WK, et al. Minocycline 1.5% foam for the topical treatment of moderate to severe papulopustular rosacea: Results of 2 phase 3, randomized, clinical trials. J Am Acad Dermatol [Internet]. 2020 May [cited 2023 Dec 14];82(5). Available from:](http://paperpile.com/b/PjMad2/GI84) <https://pubmed.ncbi.nlm.nih.gov/32004648/>

14. [Raoufinejad K, Mansouri P, Rajabi M, Naraghi Z, Jebraeili R. Efficacy and safety of permethrin 5% topical gel vs. placebo for rosacea: a double-blind randomized controlled clinical trial. J Eur Acad Dermatol Venereol. 2016 Dec;30(12):2105–17.](http://paperpile.com/b/PjMad2/Wv44)

15. [Karabulut AA, Izol SB, Eksioglu HM. A randomized, single-blind, placebo-controlled, split-face study with pimecrolimus cream 1% for papulopustular rosacea. J Eur Acad Dermatol Venereol [Internet]. 2008 Jun [cited 2023 Dec 14];22(6). Available from:](http://paperpile.com/b/PjMad2/B515) <https://pubmed.ncbi.nlm.nih.gov/18328059/>

16. [Koca R, Altinyazar HC, Ankarali H, Muhtar S, Tekin NS, Cinar S. A comparison of metronidazole 1% cream and pimecrolimus 1% cream in the treatment of patients with papulopustular rosacea: a randomized open-label clinical trial. Clin Exp Dermatol [Internet]. 2010 Apr [cited 2023 Dec 14];35(3). Available from:](http://paperpile.com/b/PjMad2/9UN2) <https://pubmed.ncbi.nlm.nih.gov/19594764/>

17. [Koçak M, Yağli S, Vahapoğlu G, Ekşioğlu M. Permethrin 5% cream versus metronidazole 0.75% gel for the treatment of papulopustular rosacea. A randomized double-blind placebo-controlled study. Dermatology. 2002;205(3):265–70.](http://paperpile.com/b/PjMad2/VoI2)

18. [Luger T, Peukert N, Rother M. A multicentre, randomized, placebo-controlled trial establishing the treatment effect of TDT 068, a topical formulation containing drug-free ultra-deformable phospholipid vesicles, on the primary features of erythematotelangiectatic rosacea. J Eur Acad Dermatol Venereol. 2015 Feb;29(2):283–90.](http://paperpile.com/b/PjMad2/EoLz)

19. [Maddin S. A comparison of topical azelaic acid 20% cream and topical metronidazole 0.75% cream in the treatment of patients with papulopustular rosacea. J Am Acad Dermatol. 1999 Jun;40(6 Pt 1):961–5.](http://paperpile.com/b/PjMad2/YHEV)

20. [Miyachi Y, Yamasaki K, Fujita T, Fujii C. Metronidazole gel (0.75%) in Japanese patients with rosacea: A randomized, vehicle-controlled, phase 3 study. J Dermatol. 2022 Mar;49(3):330–40.](http://paperpile.com/b/PjMad2/UtJq)

21. [Mostafa FF, El Harras MA, Gomaa SM, Al Mokadem S, Nassar AA, Abdel Gawad EH. Comparative study of some treatment modalities of rosacea. J Eur Acad Dermatol Venereol. 2009 Jan;23(1):22–8.](http://paperpile.com/b/PjMad2/dh37)

22. [CTG Labs - NCBI [Internet]. [cited 2023 Dec 14]. Available from:](http://paperpile.com/b/PjMad2/bRvm) <https://clinicaltrials.gov/study/NCT01174030>

23. [Phase 3 Efficacy and Safety Study of CD07805/47 Topical Gel in Subjects With Facial Erythema Associated With Rosacea [Internet]. [cited 2023 Dec 14]. Available from:](http://paperpile.com/b/PjMad2/zo8G) <https://clinicaltrials.gov/ct2/show/NCT01355471>

24. [Efficacy and Safety of AGN-199201 in Patients With Persistent Erythema Associated With Rosacea [Internet]. [cited 2023 Dec 14]. Available from:](http://paperpile.com/b/PjMad2/nhRu) <https://clinicaltrials.gov/ct2/show/NCT02131636>

25. [Safety and Efficacy of Oxymetazoline HCl Cream 1.0% in Patients With Persistent Erythema Associated With Rosacea [Internet]. [cited 2023 Dec 14]. Available from:](http://paperpile.com/b/PjMad2/GqlU) <https://clinicaltrials.gov/ct2/show/NCT02132117>

26. [A Study to Determine Safety and Efficacy of B244 in Subjects With Mild to Moderate Rosacea [Internet]. [cited 2023 Dec 14]. Available from:](http://paperpile.com/b/PjMad2/Xn4o) <https://clinicaltrials.gov/ct2/show/NCT03590366>

27. [Sauder D, Miller R, Gratton D, Danby W, Griffiths C, Phillips S. The treatment of rosacea: the safety and efficacy of sodium sulfacetamide 10% and sulfur 5% lotion (Novacet) is demonstrated in a double-blind study. J Dermatolog Treat. 1997 Jan 1;8(2):79–85.](http://paperpile.com/b/PjMad2/6pH7)

28. [Serdar ZA, Yaşar Ş. Efficacy of 1% terbinafine cream in comparison with 0.75% metronidazole gel for the treatment of papulopustular rosacea. Cutan Ocul Toxicol. 2011 Jun;30(2):124–8.](http://paperpile.com/b/PjMad2/B7iZ)

29. [Stein-Gold L, Kircik LH, Draelos ZD, Werschler P, DuBois J, Lain E, et al. WITHDRAWN: Efficacy and safety of topical oxymetazoline cream 1.0% for treatment of persistent facial erythema associated with rosacea: findings from the 2 phase 3, 29-day, randomized, controlled REVEAL trials. J Am Acad Dermatol [Internet]. 2018 Jan 31; Available from:](http://paperpile.com/b/PjMad2/D6Mz) <http://dx.doi.org/10.1016/j.jaad.2018.01.028>

30. [Tan JKL, Girard C, Krol A, Murray HE, Papp KA, Poulin Y, et al. Randomized placebo-controlled trial of metronidazole 1% cream with sunscreen SPF 15 in treatment of rosacea. J Cutan Med Surg. 2002 May 13;6(6):529–34.](http://paperpile.com/b/PjMad2/rr4y)

31. [Wang GJ, Gao XY, Wu Y, He HQ, Yu Y, Qin HH, et al. Evaluation of the efficacy and tolerance of artemether emulsion for the treatment of papulopustular rosacea: a randomized pilot study. J Dermatolog Treat. 2019 Dec;30(8):809–12.](http://paperpile.com/b/PjMad2/w1B1)

32. [Wang B, Huang Y, Tang Y, Zhao Z, Shi W, Jian D, et al. Paroxetine is an effective treatment for refractory erythema of rosacea: Primary results from the Prospective Rosacea Refractory Erythema Randomized Clinical Trial. J Am Acad Dermatol. 2023 Jun;88(6):1300–7.](http://paperpile.com/b/PjMad2/JTVA)

33. [Wei D, Hamblin MR, Wen X. A randomized, controlled, split-face study of topical timolol maleate 0.5% eye drops for the treatment of erythematotelangiectatic rosacea. J Cosmet Dermatol. 2021 Dec;20(12):3968–73.](http://paperpile.com/b/PjMad2/x8Qy)
